# MCBK 2022 Lightning Round Abstracts

**DOI:** 10.1002/lrh2.10375

**Published:** 2023-07-03

1. Alper, Brian: Fast Evidence Interoperability Resources (FEvIR) Platform to mobilize Computable Biomedical Knowledge (CBK)
2. Alper, Brian: Specifying a Common Metadata Framework to mobilize CBK
3. Blobel, Bernd: Advancing health ecosystems from the data exchange to the knowledge sharing paradigm
4. Dehnbostel, Joanne: Converting MEDLINE and ClinicalTrials.gov to FHIR
5. Dehnbostel, Joanne: Scientific Evidence Code System (SEVCO) as a standard terminology
6. Du, Jian: A PICO‐based knowledge graph for representing clinical evidence reported in ClinicalTrials.gov
7. Dushyanthen, Sathana: Designing interprofessional education to Foster Learning Health Systems
8. Flynn, Allen: Lessons learned going for CBK FAIRness
9. Goyal, Pawan: The Emergency Quality Network (E‐QUAL): Transforming quality improvement in emergency medicine
10. Heigl, Michaela: NICE Collaborathon—Exploring implementation of computable knowledge standards
11. Jacob, George: Free and open‐source solutions to using BPMN for computable CPG artifacts
12. Kong, Guilan: An evidential reasoning‐based knowledge‐and‐data driven modeling methodology for clinical decision support
13. Korom, Robert: Primary care in Kenya: The ideal setting for CDSS implementation
14. Ljazouli, Saad: Knowledge sharing via reusable code for N3C observational database Studies
15. Mayya, Vaishakhi: Extraction and retrieval of critical treatment flow and intervention data from EHR: iCare prototype system
16. Richardson, Joshua: A survey of computable biomedical knowledge repositories: Developing a desiderata for conveying trust
17. Rubin, Ken: The criticality of care processes as computable artifacts
18. Strasberg, Howard: Mobilizing knowledge conclusions with CDS Hooks
19. Swain, Deborah: Sustainable open educational resources for computable biomedical knowledge leaders
20. Tong, Beibei: Clinical prediction models for knee pain in patients with knee osteoarthritis: A systematic review and critical appraisal
21. Van Woensel, William: Operationalizing text‐based clinical practice guidelines as computable decision support on blockchain using code generation
22. Wales, Ann: The Right Decision Service—National decision support for Scotland
23. Wu, Jingyi: KGrid‐CN, A platform for scaling computable biomedical knowledge across practice in China

## Fast Evidence Interoperability Resources (FEVIR) Platform to mobilize CBK

Brian S. Alper, Computable Publishing LLC, Scientific Knowledge Accelerator Foundation. balper@computablepublishing.com

Joanne Dehnbostel, Computable Publishing LLC, Scientific Knowledge Accelerator Foundation. jdehnbostel@computablepublishing.com

Khalid Shahin Computable Publishing LLC, Scientific Knowledge Accelerator Foundation. kshahin@computablepublishing.com

Fast Healthcare Interoperability Resources (FHIR) is a standard describing data formats for exchanging electronic health records. FHIR is highly effective for mobilization of patient‐specific computable healthcare knowledge, but similar solutions have not been developed for community knowledge such scientific research and clinical practice guidance, until now.

Extension of FHIR to Evidence‐Based Medicine (EBMonFHIR) is providing a standard to mobilize evidence and guidance. FHIR Resources have been created for exchange of Citation, Evidence, EvidenceVariable, EvidenceReport, ResearchStudy, and ArtifactAssessment (to provide comments, ratings and classifiers for any other knowledge artifact). The Fast EVIDENCE Interoperability Resources (FEvIR) Platform is freely available at https://fevir.net and supports the creation and viewing of computable biomedical knowledge in standard form, using FHIR JSON where specified and FHIR‐like JSON where needed as we further develop the FHIR standard. Resources (in FHIR R5 JSON) currently on the FEvIR Platform include ActivityDefinition, ArtifactAssessment, Bundle, Citation, CodeSystem, Consent, Evidence, EvidenceReport, EvidenceVariable, Group, Organization, Practitioner, PractitionerRole, Questionnaire, ResearchStudy, ResearchSubject, StructureDefinition, and ValueSet.

The FEvIR Platform is open for viewing resources without login or registration. Signing in is free, as simple as using Google account login, and is required to create content on the FEvIR Platform as the person who creates the content is the only one with edit rights to that content.

The FEvIR Platform has 13 Viewer Tools that provide human‐friendly displays of FHIR Resources that include outline representation of the JSON and/or specialized views based on the resource type. The FEvIR Platform has eight builder tools that enable creation of a FHIR Resource without any working knowledge of FHIR or JSON.

The FEvIR Platform has three Converter Tools (MEDLINE‐to‐FEvIR, ClinicalTrials.gov‐to‐FEvIR, and FEvIR‐to‐ClinicalTrials.gov) that facilitate interoperable data exchange between systems.

The FEvIR Platform has five Specialized Tools (My Ballot, Portal View, Recommendations Table Viewer, Risk of Bias Assessment Tool, and Risk of Bias Assessment Reader) for organized creation and viewing across resources in context‐relevant combinations.

The FEvIR Platform is used to support the COVID‐19 Knowledge Accelerator (COKA). COKA is an open, virtual group to accelerate identifying, processing, and disseminating knowledge (about COVID‐19 but could be applied to any scientific knowledge). COKA efforts inform and develop standards, terminologies, and tools for computable expression of evidence and guidance. You can participate through any of 12 open virtual weekly meetings. Details at https://tinyurl.com/COKA2021

## Specifying a Common Metadata Framework to mobilize CBK

Brian S. Alper, Computable Publishing LLC (and Scientific Knowledge Accelerator Foundation). balper@computablepublishing.com

Joanne Dehnbostel, Computable Publishing LLC (and Scientific Knowledge Accelerator Foundation). jdehnbostel@computablepublishing.com

Khalid Shahin, Computable Publishing LLC (and Scientific Knowledge Accelerator Foundation).

kshahin@computablepublishing.com

Standards for data exchange greatly advance the mobilization of CBKs. Consider seven different systems, each with their own data format. Converting data from one system to another for each pairing would require creating 42 different converter tools. Using a “hub and spoke” model with a standard form for data exchange, this goal is reached with 14 converter tools: 7 to convert to the standard form and 7 to convert from the standard form for each of the 7 systems.

The Fast Evidence Interoperability Resources (FEvIR) Platform is a hub for data exchange for scientific evidence and guidance that uses a standard (Fast Healthcare Interoperability Resources [FHIR]) extended for Evidence‐Based Medicine related knowledge. Converter tools between any system and the FEvIR Platform will enable interoperable data exchange to mobilize CBKs.

An MCBK Standards Work Group subgroup published “Categorizing metadata to help mobilize computable biomedical knowledge” (https://doi.org/10.1002/lrh2.10271), which defined 13 categories of metadata most relevant to making CBKs findable, accessible, interoperable, reusable, and trustable (FAIR+T): type, domain, purpose, identification, location, CBK‐to‐CBK relationships, technical, authorization and rights management, provenance, evidential basis, evidence from use metadata, preservation, and integrity metadata.

A COVID‐19 Knowledge Accelerator (COKA) Common Metadata Framework Working Group created 129 data element structure definitions to specify metadata for 12 of these categories (all but preservation). Each data element is specified with an element name, a datatype, whether the element is required, whether the element can contain multiple values, and whether the element is a container that includes data elements.

Two crosswalks matching data elements across dataset schemas were created by the Research Data Alliance—one mapping 772 sets of data elements across 17 dataset schemas and one mapping 52 sets of data elements across 20 dataset schemas. The COKA Common Metadata Framework Working Group mapped both crosswalks to structures in the Common Metadata Framework, occasionally adjusting the Common Metadata Framework specification to accommodate the data structures in the crosswalks.

Facing a series of compound spreadsheets and recognizing the inefficiency for scaling to support many more dataset schemas, we invented a SchemaElement StructureDefinition to provide a common reusable structure for representation of a data element structure definition and mapping it to a data element structure in a different dataset schema.

Data conversion tools on the FEvIR Platform (ClinicalTrials.gov‐to‐FEvIR Converter and RIS‐to‐FEvIR Converter so far) are being used to inform the development of the Common Metadata Framework and to show how the Common Metadata Framework will facilitate the efficiency of creating additional data conversion tools to support interoperable data exchange between systems using different dataset schemas.

The Common Metadata Framework effort is conducted through the COVID‐19 Knowledge Accelerator (COKA). COKA is an open, virtual group to accelerate identifying, processing, and disseminating knowledge (about COVID‐19 but could be applied to any scientific knowledge). COKA efforts inform and develop standards, terminologies, and tools for computable expression of evidence and guidance. You can participate through any of 12 open virtual weekly meetings. Details at https://tinyurl.com/COKA2021

## Advancing health ecosystems from the data exchange to the knowledge sharing paradigm

Prof. Dr. habil. Bernd Blobel, FACMI, FACHI, FHL7, FEFMI, FIAHSI, University of Regensburg.

bernd.blobel@klinik.uni-regensburg.de

Healthcare and social care systems around the globe are currently undergoing massive organizational, technological and methodological paradigm changes. Regarding the methodology paradigms change in medicine, we experience the transformation from an empirical, phenomenological through an evidence‐based, person‐centered, and personalized up to a 5P Medicine approach. 5P Medicine stands for personalized, preventive, predictive, participative precision medicine. This approach requires the scientifically, interdisciplinary justification of the individual's status in its personal, environmental, social, occupational, and behavioral context for understanding the pathology of disease and properly managing the comprehensive care process. This requires close cooperation of experts from different domains using their perspectives, objectives, languages, and methodologies from their knowledge spaces. We need a sophisticated approach to represent and manage the health and social care ecosystem system, based on universal type theory, universal logic, philosophy, systems, and language theory. The development process has to follow the Rational Unified Process for IT systems development, standardized at ISO 10746 Reference Model Open Distributed Processing. As the representation languages for modeling and specifying appropriate solutions necessarily move toward higher expressivity with increasing restrictions to special structures but also less generative power and completeness, the deployment of good modeling practices is inevitable. Contrary to the current praxis of focusing on the processable knowledge representation (KR), we have to start with the representation of the epistemological knowledge of the involved domains and its correct formalization to transform the outcome into the processable KR. That way, interoperability advances from data exchange through information sharing, process management, knowledge management to interdisciplinary knowledge space management, thereby also acknowledging individual skills and experiences. In other words, we advance from data exchange to knowledge sharing. Our solution is an architecture‐centric, system‐theoretical, ontology‐based, and policy‐driven model and framework for a system of systems, meanwhile standardized as ISO 23903 Health Informatics—Interoperability and Integration Reference Architecture. The model presents the involved domains, the systems’ components composition/decomposition, and the systems development process represented by its ISO 10746 views. The real‐world domains are formally represented by the corresponding domain ontologies guided by top‐level ontologies, while the IT views are represented by IT ontologies from business process modeling through information and data modeling up to implementable artifacts. Beyond the intended business system and its processes, the system model may also imply behavioral aspects defined by systems policies such as security and privacy rules, and ethical principles, represented by corresponding ontologies we have meanwhile standardized. The presented solution enables interoperability and integration between different scientific domains and disciplines, but also between different system components such as models, specifications or objects at any development process viewpoint. Formally approved in 2021, ISO 23903 is already used in a series of ISO, IEC, CEN, OMG, IEEE, and HL7 standards and projects around the globe.

## Converting MEDLINE and ClinicalTrials.gov to FHIR

Joanne Dehnbostel, Computable Publishing LLC and Scientific Knowledge Accelerator Foundation. jdehnbostel@computablepublishing.com

Brian S. Alper, Computable Publishing LLC and Scientific Knowledge Accelerator Foundation. balper@computablepublishing.com

Khalid Shahin, Computable Publishing LLC and Scientific Knowledge Accelerator Foundation.

kshahin@computablepublishing.com

Technical standards advance mobilization of CBKs by facilitating interoperable data exchange. Consider seven different systems managing data about scientific studies, each with their own data format. If one desired to convert data from one system to another for each pairing, it would require creation of 42 different converter tools. Using a “hub and spoke” model, if one created a standard form for data exchange, it could meet the same goal with 14 converter tools, 7 to convert to the standard form and 7 to convert from the standard form for each of the 7 systems.

The Fast Evidence Interoperability Resources (FEvIR) Platform (https://fevir.net) is a hub for data exchange for scientific evidence and guidance which uses a technical standard based on extending Fast Healthcare Interoperability Resources (FHIR) for evidence‐based medicine‐related knowledge.

Conversion between any system and the FEvIR Platform will enable realization of the interoperable data exchange to mobilize CBKs. The FEvIR Platform provides access to an array of tools to create and view scientific knowledge in standards‐based, machine‐interpretable units called Resources.

Computable Publishing®: MEDLINE‐to‐FEvIR Converter https://fevir.net/medlineconvert converts data in PubMed MEDLINE XML to a FHIR Citation Resource in JSON format.

The resulting FHIR Citation Resource will have content (when applicable data is found) including title, abstract, identifiers (PMID, DOI, pii), related identifiers (ClinicalTrials.gov NCTId), author list, classifiers from MEDLINE including Medical Subject Headings (MeSH), URLs (abstract on PubMed and DOI‐based link), publication details, and related articles (cited articles, comments, corrections and retractions).

You can clone the Citation Resource and use Computable Publishing®: Citation Builder/Viewer to add additional data including classifiers. Creation of a citation produces a Computable Publishing Style citation summary that can be used as a citation in a list of references.

Computable Publishing®: ClinicalTrials.gov‐to‐FEvIR Converter https://fevir.net/ctgovconvert converts data from the protocol and results sections of ClinicalTrials.gov JSON for a selected NCTID to FEvIR Resources in FHIR JSON format.

The resulting FHIR Resources (depending on the data available) may include ResearchStudy (the primary study record), Citation (using the MEDLINE converter mentioned above, for any articles associated with the study record), EvidenceVariable (for the eligibility criteria and study outcomes), Organization or Location (for study sites), Practitioner or PractitionerRole or Organization (for sponsors, investigators and collaborators), Group (for the study group), Evidence (results), and EvidenceReport (collection of results). An API will soon be available to produce FHIR resources using this conversion tool on the ClinicalTrials.gov website.

The FEvIR Platform is open for viewing without login or registration. Sign‐in is free, Google login is required to create content on the FEvIR Platform so the person who creates the content maintains edit rights.

The FEvIR Platform is used to support the COVID‐19 Knowledge Accelerator (COKA), an open, virtual group to accelerate identifying, processing, and disseminating knowledge. COKA efforts inform and develop standards, terminologies, and tools for computable expression of evidence and guidance. You can participate in 12 open virtual weekly meetings. Details at https://tinyurl.com/COKA2021.

## Scientific Evidence Code System (SEVCO) as a standard terminology

Joanne Dehnbostel, Computable Publishing LLC.

jdehnbostel@computablepublishing.com

Brian Alper, Computable Publishing LLC.

balper@computablepublishing.com

Khalid Shahin, Computable Publishing LLC.

kshahin@computablepublishing.com

In many domains standard terminologies facilitate unambiguous communication. However, there are no globally accepted and adopted standard terminologies for reporting scientific knowledge. A global effort started in 2020 to define standard terminologies (in the form of code systems) for 4 scientific knowledge concepts: Study Design, Risk of Bias, Statistic Type, and Statistical Model.

A Code System Development Protocol was created in September 2020 to support global development of terminologies for exchange of scientific evidence. Initial steps of the protocol included (1) assembling expert working groups with people from more than 25 countries, (2) identifying 23 commonly used tools and systems for which standard terminology would be useful, (3) drafting 368 non‐redundant concepts to become display terms for four code systems (Statistic Type, Statistic Model, Study Design, Risk of Bias), (4) identifying 27 ontologies with related terms and definitions, and (5) mapping available terms and definitions for the draft concepts. (Alper BS, Dehnbostel J, Afzal M, Subbian V, Soares A, Kunnamo I, Shahin K, McClure RC, For the COVID‐19 Knowledge Accelerator (COKA) Initiative. Making Science Computable: Developing code systems for statistics, study design, and risk of bias. Journal of Biomedical Informatics 2021 Mar;115:103685. https://doi.org/10.1016/j.jbi.2021.103685).

We consolidated these efforts into a single Scientific Evidence Code System (SEVCO), developed tooling to facilitate the effort (including Computable Publishing®: CodeSystem Builder/Viewer and Computable Publishing®: My Ballot), and streamlined the protocol (Alper BS, Dehnbostel J, Lehmann H, Whaley P, Wilkins KJ, Tufte J, Yurk RA, Ojha N, Afzal M. For the COVID‐19 Knowledge Accelerator (COKA) Initiative. Scientific Evidence Code System Development Protocol. Created November 16, 2021. Last revised December 8, 2021. Available at: https://tinyurl.com/SEVCOprotocol).

The developing Scientific Evidence Code System (SEVCO) is available for open viewing at https://fevir.net/resources/CodeSystem/27270#TOP and anyone can comment on any term. Participation in deliberation of the terminology is also open to anyone. You can self‐select to join the SEVCO Expert Working Group at https://fevir.net/resources/Project/27845 and as terms are opened for voting you can vote Yes or you can vote No and provide a comment for what change is needed. Terms are passed when achieving 100% agreement with at least five votes, and negative votes lead to discussion and modification.

As of May 27, 2022, there are 563 terms identified in SEVCO and 206 (37%) have unanimous approval.

When completed, the code system will facilitate identifying, processing, and reporting research results and the reliability of those results. More efficient and detailed scientific communication will reduce cost and burden and improve health outcomes, quality of life, and patient, caregiver, and healthcare professional satisfaction.

SEVCO is developed by the COVID‐19 Knowledge Accelerator (COKA). COKA is an open, virtual group to accelerate identifying, processing, and disseminating knowledge (about COVID‐19 but could be applied to any scientific knowledge). COKA efforts inform and develop standards, terminologies, and tools for computable expression of evidence and guidance. You can participate through any of 12 open virtual weekly meetings. Details at https://tinyurl.com/COKA2021

## A PICO‐based knowledge graph for representing clinical evidence reported in ClinicalTrials.gov

Jian Du, National Institute of Health Data Science, Peking University.

dujian@bjmu.edu.cn

Compared to unstructured representation of clinical evidence in bibliographic databases such as PubMed, structured results data available in clinical trial registries, for example, Clinicaltrials.gov may be more timely, complete, and accessible. However, these data remain underutilized. In our work, we extended the idea of “computable evidence synthesis” by presenting PICO element and trial results (ie, the effects of interventions on outcomes) reported in registries in a standardized format with biomedical ontologies, such as UMLS and OHDSI CDM vocabularies. While ClinicalTrials.gov provides structured data download (CSV format), the key information in PICO framework is not present, such as (1) distinguishing Intervention (I) from Comparison (C), (2) the combination of drugs as Intervention (I), and (3) the detailed description of Outcomes (O) and their statistical measures. Fortunately, the U.S. National Library of Medicine (NLM) distributes ClinicalTrials.gov in eXtensible Markup Language (XML)‐formatted files for each study. We used self‐compiled software tools to parse the XML data files and extract PICO and the *P*‐value for a specific outcome. The relationship between an investigated intervention and the outcome of interest is judged by *P*‐value and/or 95% Confidence Interval (CI). If “*P*‐value greater than 0.05,” and/or “the lower CI smaller than 1 and the upper CI greater than 1,” it indicates to “has no difference,” while “*P*‐value less than 0.05” and/or “both the lower and the upper CI smaller than 1 or greater than 1” relates to “has significant difference.” We then loaded the content into a graph database to demonstrate the entities and their relations and make PICO elements and the Effects easy to query. Compared with publications in bibliographic databases, these data include negative and positive outcomes. Batch queries can be acquired in a Graph Database built by Neo4j through Cypher language. The knowledge graph is expected to give a whole picture on reported results of clinical trials and provided computable format for evidence synthesis.

## Designing interprofessional education to Foster Learning Health Systems

Sathana Dushyanthen, Centre for Digital Transformation of Health, University of Melbourne. sathana.dushyanthen@unimelb.edu.au

Meg Perrier, Centre for Digital Transformation of Health, University of Melbourne. m.perrier@unimelb.edu.au

Wendy Chapman, Centre for Digital Transformation of Health, University of Melbourne. wendy.chapman@unimelb.edu.au

Meredith Layton, Centre for Digital Transformation of Health, University of Melbourne. meredith.layton@unimelb.edu.au

Kathleen Gray, Centre for Digital Transformation of Health, University of Melbourne.

kgray@unimelb.edu.au

Kayley Lyons, Centre for Digital Transformation of Health, University of Melbourne. kayley.lyons@unimelb.edu.au

**Introduction:** The “60‐30‐10” challenge is one of EPIC proportions within healthcare, and continues to worsen, with wastage, process inefficiencies and non‐optimal patient care and safety practices. Learning Health Systems (LHS) have the potential to utilize biomedical health data in real time, through rapid and continuous cycles of data interrogation, implementing insights to practice, feedback, and practice change. However, there is a lack of an appropriately skilled inter‐disciplinary, informatics workforce that are able to leverage computable biomedical knowledge to design innovative solutions. Therefore, there is a need to develop tailored professional development training to foster skilled interdisciplinary learning communities in the healthcare workforce in Australia.

**Methods:** In order to teach LHS principles, we have developed multiple educational programs of work including a professional development short course and a year‐long Academy Fellowship program. We developed a wholly online short course, open to interdisciplinary professionals working in digital health in the healthcare sector. In a flipped classroom model, participants undertook 2 hours of pre‐class learning online and then attended 2.5 hours of live synchronous learning in interactive Zoom workshops, weekly, for 13 weeks. We further applied this short course to our LHS Academy whereby the Fellows completed this course as part of their foundational coursework before undertaking 8 months of LHS project‐based learning.

In order to evaluate the utility of program we undertook a mixed methods evaluation consisting of pre and post‐surveys with ratings scales for usefulness, engagement, value, and applicability for various aspects of the course. Participants also completed identical measures of self‐efficacy pre and post, with a scale that was mapped to specific skills and tasks that should have been achievable following each of the topics covered. Post‐course participants were invited to participate in a semi‐structured interview process and surveys to elaborate on survey questions and dive deeper into themes around utility, future applicability, barriers, recommendations, and identity in digital health.

**Results:** From the evaluation, it was evident that participants found the teaching model engaging, useful, valuable and applicable to their work and LHS projects. In the self‐efficacy component, we observed a significant increase in perceive confidence for all topics, when comparing pre and post‐course ratings. Overall, it was evident that the program gave participants a framework to organize their knowledge; a common understanding and shared language to converse with other disciplines; changed the way they perceived their role and the possibilities of data and technologies; and finally, LHS provided a toolkit to operate from. For the LHS Academy Fellows, there was also a notable increase in the number of fellows perceiving themselves as leaders at the end of their foundational coursework.

**Conclusion:** We present examples of LHS‐specific education programs as means of educating the health workforce to adopt the LHS model into standard practice. However, it is evident that such a movement will require a global and coordinated effort with significant training for the workforce to be able to understand and utilize data effectively, to improve practice.

## Lessons learned going for CBK FAIRness

Allen Flynn, University of Michigan.

ajflynn@med.umich.edu

Zach Landis‐Lewis, University of Michigan.

zachll@umich.edu

Peter Boisvert, University of Michigan.

pboisver@med.umich.edu

This lightning talk shares lessons learned while exploring new methods of packaging CBK artifacts to make them FAIR (ie, findable, accessible, interoperable, and reusable). We highlight implementation choices that leverage the decentralized technologies of the World Wide Web (WWW). We view packaged CBK artifacts as a heterogeneous class of Digital Objects that are, “transient assemblies of functions, information items, or components spread over information infrastructures and the Internet, a condition that sets them strongly apart from physical objects” (due to their malleability and interactivity).^1^, ^2^ To make CBK artifacts stable and usable, our packages add content alongside CBK, including identifiers, pointers, documentation, tests, and metadata.^3^ Here, we share the results of several implementation choices.

LESSON 1—Choose identifier systems intentionally

To find CBK artifacts on the WWW, new persistent unique identifiers (PUIDs) must be minted and assigned to each one. We appreciate the Handle System.^4^, ^5^ However, we have instead implemented Archival Resource Keys (ARKs), an identifier system born at the California Digital Library and sustained by the ARK Alliance.^6^, ^7^ ARKs offer local control over identifier shoulders and subnamespaces.^8^ We use ARK subnamespaces to reflect our evolving ontology of CBK artifact parts.^9^ In this way, ARKs enable a standardized pointer scheme for the subcomponents of our packaged CBK artifacts.

LESSON 2—Leverage resolver‐registries to increase access

To access CBK artifacts with persistent identifiers on the WWW, we have explored how to take advantage of the Name‐to‐Thing ID resolver (N2T.net).^9^ To increase access, identifier resolver technology like N2T.net provides lookup and HTTP redirection to various CBK artifact servers and repositories.

LESSON 3—Use service descriptions to achieve interoperability

To interoperate with CBK artifacts via common webservices, we incorporate machine‐readable service descriptions into our packages. Our initial approach uses service descriptions conforming to the OpenAPI 3x standard for RESTful webservices.^10^, ^11^ Using these service descriptions and some homegrown technology for activating CBK artifacts,^12^ we gain interoperability with the Swagger Editor. We have demonstrated how webservices backed by CBK artifacts can be rapidly deployed on any server, enabling horizontal scaling while supporting localized webservice operation.

LESSON 4—Package versioning and provenance metadata for reusability

To make CBK artifacts reusable, versioning and provenance metadata are required. Whole artifacts and their subcomponents are subject to versioning by various methods. We use GitHub for versioning and provenance. While GitHub is proprietary, its underlying artifact management approach, Git, is open‐source. We consider our CBK artifacts to be “under management” when they have been stored, versioned, and released in GitHub. To further enable reusability, we also use GitHub for sharing licenses and user documentation about each CBK artifact.

We are moving toward treating CBK artifacts as first‐class items in a linked‐data world. We have tried ARK IDs for finability, N2T.net for accessibility, service descriptions in for interoperability, and GitHub for versioning and provenance. Contact us for more details about these and other implementation choices we have explored.

## The Emergency Quality Network (E‐QUAL): Transforming quality improvement in emergency medicine

Pawan Goyal, American College of Emergency Physicians.

pgoyal@acep.org

Melissa Finch, American College of Emergency Physicians.

mfinch@acep.org

Cindy Mendez‐Hernandez, American College of Emergency Physicians. cmhernandez@acep.org

Dhruv Sharma, American College of Emergency Physicians.

dsharma@acep.org

Arjun K. Venkatesh, Yale School of Medicine.

arjun.venkatesh@yale.edu

**Problem:** The call for quality improvement (QI) and practice transformation is increasing attention on the practice of emergency medicine at a national level. The Emergency Quality Network (E‐QUAL) identified six focus areas with clinical salience for physician engagement and practice transformation: sepsis, avoidable imaging, chest pain, opioid harm reduction, stroke care, and atrial fibrillation.

**Measurement:** E‐QUAL enrolls emergency departments (EDs) in year‐long initiatives in which the ED champion interacts with the E‐QUAL virtual portal to report on local quality improvement activities. These include completing the online portal activities, engaging eligible clinicians in local QI projects, utilizing educational toolkits, webinars, podcasts, and submitting benchmarking data.

**Analysis:** E‐QUAL participants provide data in three forms: Survey (eg, Readiness, Capabilities, and Best Practices’ Assessments), Quality Benchmarking (ie, Metrics‐driven Chart Abstract), and Viewing Educational Content (eg, webinars, podcasts, and continuing medication education). We report descriptive statistics on ED demographics, clinician enrollment in E‐QUAL overall and by collaborative, engagement in annual QI, and performance in EM QI metrics across several years. Due to data being self‐reported, analytics are limited to descriptive statistics.

**Implementation:** Best practices are deployed in various formats such as: toolkits, webinars, workflows, and subject matter work groups to refine QI strategies to be utilized in both rural and urban ED sites. The breadth of E‐QUAL's reach nationwide provides an elevated view of the challenges impacting emergency medicine.

**Results/Discussion:** The rapid growth of the E‐QUAL network shows broad interest among the emergency medicine community in engaging in quality improvement to deliver higher‐value care. Through engagement of emergency clinicians and leveraging of EDs to improve key clinical outcomes, E‐QUAL has demonstrated 25 000 lives saved from better sepsis care, 30 000 fewer patients harmed due to ionizing radiation from unnecessary imaging studies, $55 million saved from fewer avoidable imaging and hospitalizations, along with a 35% reduction of opioid prescribing practices.

## NICE Collaborathon—Exploring implementation of computable knowledge standards

Michaela Heigl, The National Institute for Health and Care Excellence.

michaela.heigl@outlook.com

Leo Jared, The National Institute for Health and Care Excellence.

leo.jared@nhs.net

Philip Scott, University of Wales Trinity Saint David.

philip.scott@uwtsd.ac.uk

The National Institute for Health and Care Excellence (NICE) is the authoritative source of evidence‐based clinical knowledge for the NHS in England and Wales. Its five‐year strategy has identified the need for “a shift from ‘words’ to ‘data’ and to a more modular approach to recommendations” (NICE, 2021). The strategy identifies that a key success factor is “having guideline recommendations produced in an interactive, digitalised format.”

NICE has worked closely with the MCBK UK group since it was formed in 2019 (Mitchell, 2020). In 2020, NICE established a Content Advisory Board to help it move toward the strategic digital aims.

The MCBK UK group proposed a ‘collaborathon’ (or ‘hack day’) to activate industry involvement and understand vendor capability and willingness to adopt international standards for computable knowledge, based on learning from a similar event run for Health Data Research UK in 2020 (Scott, 2021).

The aim of the collaborathon is to explore a range of technical approaches to transition NICE toward production of natively digital content for education and standards‐based computable decision support. The objectives are (1) to bring together relevant stakeholders in the ecosystem for natively digital NICE content, (2) To demonstrate a selection of: use cases, knowledge types, usage patterns, integration options, decision types, and technical standards, and (3) to educate the stakeholder community, generate new ideas and assess the relative merits of a variety of methods.

The event will feature several clinical scenarios, potentially: antimicrobial prescribing, urgent referrals for suspected cancer, diabetes medication, or rare disease diagnosis.

The plan is to have two in‐person collaborathons, in November 2022 and February 2023. Both events will be multidisciplinary, but with some tasks that are primarily clinical and some that are primarily technical. The primarily clinical tasks at the first event are to decompose selected NICE narrative into useful tagged components and to note any common principles for content decomposition across specialty topics. The primarily technical tasks are to represent a selected NICE narrative recommendation or structured pathway in a fully specified logical model using CQL or BPM+. At the second events the planned tasks are to (1) implement a CDS Hook or lookup for the tagged fragments identified in the first meeting in a simulated EPR or app and (2) implement a prepared logical model (CQL or BPM+) in a simulated EPR or app.

Before the first collaborathon, we will develop online tutorials on FHIR, CQL, and BPM+ for participants and clarify the clinical use cases. Before the second collaborathon, we need to build the requisite infrastructure: FHIR servers, CQL engine, BPM+ engine, and synthetic data.

We are recruiting clinicians to participate along with invited vendor teams. We look forward to sharing our learning with the global MCBK community.

Mitchell, A. (2020). A NICE perspective on computable biomedical knowledge. BMJ Health & Care Informatics, 27(2), e100126. https://doi.org/10.1136/bmjhci-2019-100126

NICE. (2021). NICE strategy 2021 to 2026. https://www.nice.org.uk/about/who‐we‐are/corporate‐publications/the‐nice‐strategy‐2021‐to‐2026

Scott, P. (2021). Computable knowledge Part 2 of 2: learning from the HDR UK collaborathon—make it FAIR! Health Data Research UK. https://www.hdruk.ac.uk/news/computable-knowledge-part-2-of-2-learning-from-the-hdr-uk-collaborathon-make-it-fair/

## FREE and open‐source solutions to using BPMN for computable CPG artifacts

George P. Jacob, Chatham‐Kent Health Alliance, Adjunct Faculty Western University.

gjacob2010@gmail.com

Business Process Modeling Notation has been pioneered by the BPM‐Plus group to create computable clinical practice guidelines. However, expensive software‐as‐a‐service accounts are still required to create computable artifacts. This presents a significant barrier for widespread use of BPMN in low‐resource clinical settings. We present a free and open‐source option to author computable CPGs using Google Sheets and bpmn.io (free online BPMN modeler) in the form of shareable REST API endpoints. In addition, we demonstrate how these endpoints can be easily converted into web apps. We also show how web‐browser‐technology can be incorporated with EMRs (like Cerner and Telus PSS in Canada) to make CPGs available at the point of care for clinical decision support. In our example, computable CPGs allowed for collection of data to determine what percentage of a clinician's practice was guideline‐based. These data can then be used to create a learning health system. Our work presents a free option for health systems/clinicians to use BPMN in low‐resource settings while allowing for interoperability with multiple EMR vendors due to the use of browser technology.

## An evidential reasoning‐based knowledge‐and‐data driven modeling methodology for clinical decision support

Guilan Kong, National Institute of Health Data Science at Peking University.

guilan.kong@hsc.pku.edu.cn

With the development of artificial intelligence and the accumulation of big data in medicine, data‐driven clinical decision support systems (CDSSs) have attracted attentions of researchers in medical informatics and health data science. Meanwhile, traditional knowledge‐driven CDSSs are also in the process of rapid development. However, most data‐driven CDSSs are black‐box tools, and knowledge base construction is a challenging job in knowledge‐driven CDSSs, especially for uncertain medical knowledge representation and inference. Driven by the need of making better use of not only real‐world big data but also medical domain knowledge, we propose an Evidential Reasoning‐based Knowledge‐and‐Data driven (ERKD) modeling methodology for clinical decision support in this study.

The proposed ERKD model consists of three sub‐models: a knowledge‐driven model, a big data‐driven model, and a knowledge‐and‐data fusion model for integrating the results generated by knowledge‐driven and data‐driven models. In the knowledge‐driven model, domain knowledge is represented using belief rules, which can represent medical uncertainties by introducing new parameters including rule weight, antecedent attribute weight and belief degrees in consequents; and the inference mechanism is implemented using the evidential reasoning (ER) approach, which can reason with the parameters for uncertainty representation to produce a distributed result with combined degree of belief in each possible consequent. In the big data‐driven model, real‐world evidence is mined from real‐world big data using a generalized Bayesian method and the evidence is profiled using a belief distribution format with evidence weight, reliability, independence degree, and degree of belief in each medical outcome contained in the evidence; and the ER rule with the updated and improved ER algorithm is used to do evidence combination. In the knowledge‐and‐data fusion model, the ER approach is used to integrated the results generated by the data‐driven and knowledge‐driven models.

The proposed ERKD model takes advantages of domain knowledge and real‐world big data, and the results generated by the knowledge‐driven and data‐driven sub‐models are treated with equal importance in the knowledge‐and‐data fusion sub‐model. In addition, uncertainties in medical domain knowledge can be well represented and inferred in the ERKD model, and the big data‐driven model is not a black‐box tool here, and the real‐world evidence and the ER rule‐based evidence combination process in the big data‐driven model are explainable to clinicians. Next, we plan to apply the ERKD model to CKD complications and adverse outcomes prediction.

## Primary care in Kenya: The ideal setting for CDSS implementation

Robert Korom, Penda Health.

robert@pendahealth.com

Sarah Kiptinness, Penda Health.

sarah.kiptinness@pendahealth.com

In order for Computable Biomedical Knowledge (CBK) to positively improve health outcomes, it must fulfill the fundamental theorem of medical informatics: The computer plus the clinician must produce a better outcome for the patient than the clinician alone.

It has been challenging to demonstrate this in well‐resourced settings because baseline performance of clinicians is very high and clinical quality is generally excellent. Most clinicians are specialists in their fields, which makes it very difficult for clinical decision support to substantially enhance quality while not creating additional friction for clinicians.

In our setting in Nairobi, Kenya, we provide comprehensive primary healthcare to adults and children across 19 outpatient clinics. All clinicians are Kenyan Clinical Officers—non‐physician healthcare providers. We believe this is the ideal setting to implement clinical decision support because the baseline clinical quality is much lower than other settings due to the breadth of conditions that are commonly encountered by less specialized clinicians.

We have implemented a knowledge‐based clinical decision support system (CDSS) within our electronic health record which is used at the point of care. The CDSS spans over 300 commonly encountered complaints and diagnoses and offers recommendations including history and examination templates, laboratory investigations, imaging studies, pharmaceutical treatments, referrals, and patient education materials. Clinicians are required to act on these suggestions before closing the visit and their acceptance of guideline recommendations is tracked in real‐time at the clinic and individual clinician level. There is a closed‐loop feedback system whereby clinic managers who are clinical officers themselves review guideline adherence with their teams according to the real‐time CDSS acceptance data.

Since implementing this clinical decision support system, guideline adherence has improved from a national baseline of approximately 40% (MOH Kenya, 2018) to 82% within our organization. We have seen dramatic improvements in clinically important areas such as the provision of ORS and zinc to children with diarrheal illness. We have also seen great improvements in the standardization of antibiotic prescribing for common outpatient illnesses such as Group A Strep pharyngitis and uncomplicated urinary tract infection.

The CBK used in our decision support system is conceptually simple: It ties guideline recommendations to patient complaints and diagnoses. For example, a recommendation for a urine pregnancy test is linked to a chief complaint of amenorrhea or missed periods. When a clinician selects a diagnosis of uncomplicated urinary tract infection, a pop‐up recommends alternatives based on local antimicrobial resistance patterns.

We believe that CBK has the greatest potential to improve health outcomes in low‐resource primary care settings, and yet, there has been very little study of such efforts. Our experience suggests that a straightforward clinical decision support system implemented broadly in an outpatient primary care network can more than double clinician adherence to guideline recommendations, which ultimately ensures better outcomes for patients.

## Knowledge sharing via reusable code for N3C observational database studies

Saad Ljazouli, Palantir Technologies, Denver, CO, USA.

sljazouli@palantir.com

Alfred Jerrod Anzalone, Department of Neurological Sciences, University of Nebraska Medical Center, Omaha, NE, USA.

alfred.anzalone@unmc.edu

Harold P. Lehmann, Department of Medicine, Johns Hopkins, Baltimore, MD, USA.

lehmann@jhmi.edu

Richard A. Moffitt, Department of Biomedical Informatics, Stony Brook University, Stony Brook, NY, USA.

richard.moffitt@stonybrookmedicine.edu

Katie R. Bradwell, Palantir Technologies, Denver, CO, USA.

kbradwell@palantir.com

Johanna J. Loomba, Integrated Translational Health Research Institute of Virginia, University of Virginia, Charlottesville, VA, USA.

jjl4d@virginia.edu

We present the National COVID Cohort Collaborative (N3C) Knowledge Store, a repository of reusable code, phenotype definitions, and derived variable definitions specifically for electronic health record (EHR) analysis. Referred to as the Enclave, N3C is the largest US centralized, publicly available collection of observational patient health records to date within a highly secure repository, governed by the National Institute of Health (NIH) privacy rules. The Enclave requires tools that support hugely diverse research questions, data cleaning, and analytical workflows required for EHR data, while at the same time ensuring adherence to privacy rules and protections. To address this challenge, we created the N3C Knowledge Store to allow researcher‐to‐researcher knowledge and code sharing. The Knowledge Store currently contains 99 published “Knowledge Object” items—these Objects include custom code templates, imported external datasets, authored reports, assembled cohorts, and custom Python libraries. The custom code templates implement computable phenotypes, visualizations, and derived variables (eg, calculation of BMI). Each Knowledge Object has the following required features: author, maintainer, and documentation; and optional features: patient datasets of code output, and examples of how the code template is deployed in our platform‐dependent code workbook tool. Tags for Knowledge Object items (ie, programming language or domain of OMOP table) were developed, considering template type and when in the research life cycle the template is intended for use. We also encourage peer review of Knowledge Objects, and have enabled a review process attached to the Knowledge Store item. Details on provenance and usage—such as originating research project and consuming research project—are available for each item. Knowledge Objects have been consumed 1866 times by 127 N3C research projects to enable COVID‐19 research and are also cited by peer‐reviewed published manuscripts from those research projects. One example of a Knowledge Object success story includes the Patient Severity & Scores template. This template, which is the most used Object, generates a set of critical visits for each patient of the N3C Cohort and derives the patients COVID testing status and WHO severity for each visit. The Patient Severity & Scores Knowledge Object was consumed by 43 research projects, containing 424 researchers. A key challenge in this effort arose due to the privacy needs of the Enclave. Specifically, most analytic code refers to datasets with strictly regulated access. Therefore, successful code must be stripped of such direct access to be shared. This extra effort has historically been a barrier to complete sharing and certainly limits the sharing of the code outside the Enclave. Our experience with Knowledge Objects in a centralized environment points to real needs for making computational objects abide by FAIR principles. Future goals of the Knowledge Store include expansion of Protocol Objects—complete protocols documented by researchers to answer research questions in relation to COVID‐19 and publication of a template outside the Enclave—to encourage peer review and re‐use. The Knowledge Store has made EHR analysis more accessible and less duplicative for the 2615 researchers who have joined N3C and we continue to work on improving and augmenting this valuable resource.

## Extraction and retrieval of critical treatment flow and intervention data from EHR: iCare prototype system

Vaishakhi Mayya, University of North Carolina—Chapel Hill.

vmayya@unc.edu

Javed Mostafa, University of North Carolina—Chapel Hill.

jm@unc.edu

Computer interpretable guidelines (CIG) provide an opportunity to design clinical guidelines that can be integrated into computer systems in hospitals and provide real‐time guidance to clinicians at the point‐of‐care. In fact, use of CIGs in oncology have been shown to improve outcomes and reduce costs. However, current solutions lack integration with the underlying electronic health record (EHR) systems and therefore do not fit well with clinical workflow. This can lead to an additional burden for the clinician to search for the required information to make critical decisions. Our solution is to develop a user interface that provides the clinician with the required information directly from the EHR for breast cancer treatment. We do this by representing the guidelines as a flowchart and using the flowchart as a framework to retrieve relevant documents. By focusing on critical decision points and the information necessary to make those decisions, we can connect the CIGs with the underlying EHR system. We worked with oncologists and industry members who design clinical decision support systems to develop the system we called iCare (Intelligent Clinical‐decision‐support based on an Adaptable, Rule‐based and Extensible Platform).

## A survey of computable biomedical knowledge repositories: Developing a desiderata for conveying trust

Joshua E. Richardson, RTI International.

jrichardson@rti.org

Jodyn E. Platt, University of Michigan Medical School, Department of Learning Health Sciences. jeplatt@umich.edu

Anthony Solomonides, Research Institute, NorthShore University HealthSystem. asolomonides@northshore.org

Philip D. Walker, Annette and Irwin Eskind Family Biomedical Library and Learning Center, Vanderbilt University.

philip.d.walker@vanderbilt.edu

Philip Amara, University of Michigan Medical School, Department of Learning Health Sciences. amaphili@umich.edu

Blackford Middleton, Closed Loop, Inc.

bmiddleton1@gmail.com

Successful learning health systems require trustworthy knowledge repositories—such as those that house clinical decision support artifacts—that manage computable biomedical knowledge (CBK), sustain infrastructure, and deliver effective governance. Trustworthy data practices have been asserted in principle‐based frameworks such as FAIR (findable, accessible, interoperable, and reusable) and TRUST (Transparency, Responsibility, User Focus, Sustainability, and Technology) (Lin et al.), and might be applied to CBK and CBK repositories. Yet, more needs to be done to understand CBK knowledge repository policies, standards, and practices for promoting and using trustworthy CBK artifacts. We surveyed the policies and procedures that CBK repositories in the United States currently employ to convey trust. Our findings found general trends in governing principles and provided a basis for suggested “desiderata” with which repositories may use to convey trust in CBK.

MCBK's Trust and Policy Working Group (TPWG) conducted an online survey, identifying a convenience sample of 24 knowledge repositories based on expert knowledge and review of the field. The survey was designed to inquire about policies and procedures for conveying trust in CBK. We asked 91 questions (60 structured and 31 unstructured) about the organizations themselves as well as questions that were aligned to the “TRUST” principles for data repositories. We vetted the questions through multiple rounds with TFWG members as well as with non‐members during the 2021 MCBK Annual Conference. We conducted a quantitative analysis of data from respondents who had completed at least 40% of the survey questions to generate summary frequencies of the answers, and organized governance practices into three categories: “common,” “somewhat common,” or “uncommon.”

Thirteen of the 24 CBK representatives (54%) sufficiently completed the survey. All 13 indicated to different degrees their adherence to policies that conveyed aspects of TRUST. Policies that were more commonly addressed were policies related to: Transparency, which was conveyed by having policies pertaining to provenance, credentialed contributors, and the provision of metadata; Responsibility, repositories reportedly provided knowledge in machine‐readable formats, included implementation guidelines, and adhered to standards; and Technology, which included functions that enabled end‐users to verify, search, and filter for knowledge products. Less common were practices that addressed: User Focused procedures that enabled consumers to know about user licensing requirements or query the use of knowledge artifacts; and Sustainability, less than a majority post described their sustainability plans. Of note, it was “uncommon” for patients to reportedly play a role in repositories' governance or decision‐making processes. Based on our findings, we developed 29 desiderata, organized by the TRUST principles, that we believe will promote TRUST among the CBK repositories and catalyze maturation in the CBK ecosystem.

This, to our knowledge, is the first such survey to inquire about knowledge repository governance practices to promote trust in CBK. We intend to field another survey with an increased response rate and that reports any changes in CBK governance policies, particularly policies pertaining to patient involvement.

## The criticality of care processes as computable artifacts

Ken Rubin, Department of Veterans Affairs.

kenneth.rubin@va.gov

Shane McNamee, SmileCDR. shane.mcnamee@smiledigitalhealth.com

Theresa Cullen, Pima County Health Department. theresa.cullen@pima.gov

With medical knowledge doubling every 6 months (per an NLM assessment), the need has never been greater to advance paths to promote unfettered expression and consumption of healthcare knowledge assets as directly computable artifacts. While huge investments have been made in advancing data interoperability, their impact and utility are limited if their impact on the care process is limited. Business Process Modeling brings to workflow and care pathways the rigor and computability to advance interoperable processes.

The BPM+ Health community has developed a Practical Guide to Sharable Clinical Pathways that leverages open, standards‐based formalisms into computable models that are interoperable and sharable. This lightning session will introduce BPM+, brief the status of the community, provide a tour of the products that have been developed. It will explore the utility of BPM+ and familiarize attendees with the impacts computable processes can make in guideline adherence, accelerating institutional adoption, care quality, and quality measurement.

## Mobilizing knowledge conclusions with CDS Hooks

Howard R. Strasberg, MD, MS, Wolters Kluwer Health.

howard.strasberg@wolterskluwer.com

Knowledge to improve health care can be incorporated into clinical systems via two different methods: (1) by exchanging formal, computable knowledge artifacts for incorporation into local Health IT environments; and (2) by connecting clinical systems to cloud‐based web services such that knowledge conclusions can be presented to clinicians. CDS Hooks is an HL7 Standard for Trial Use (STU) that provides a web services specification for the latter use case. The specification leverages HL7 FHIR for the exchange of patient data and defines a set of workflow hooks to trigger calls to the web service. Common examples of hooks include “patient‐view” (opening the chart), “order‐select” (selecting an item from the order catalog), and “order‐sign” (signing fully specified orders). The CDS Hooks service returns knowledge conclusions in the form of cards, which can offer textual advice, suggest orders in an interoperable format, or recommend relevant SMART on FHIR apps.

Centralizing knowledge execution in the cloud has several advantages. First, when the knowledge needs to be updated because of changes to the underlying evidence, it only needs to be updated in one location. Local deployments of computable knowledge risk becoming out of date without proper oversight and vigilance. Second, the various clinical systems only need to implement the CDS Hooks specification for calling a cloud service rather than the perhaps more difficult task of implementing an engine that can evaluate computable knowledge artifacts. Third, it may be easier to implement certain complex statistical or machine learning models in the cloud than in local environments. Fourth, for very large knowledge bases such as drug–drug interactions, cloud solutions would eliminate the need to deploy locally and keep current potentially thousands of knowledge artifacts. Fifth, by leveraging the “feedback” feature of CDS Hooks, the cloud service can be kept informed of which suggestions were accepted by clinicians under which clinical circumstances, thereby enabling a learning feedback loop for continuous improvement of the CDS service.

The combination of CDS Hooks and FHIR provides an unprecedented opportunity to mobilize knowledge conclusions to improve the quality of health care globally.

## Sustainable open educational resources for computable biomedical knowledge leaders

Deborah E. Swain, NC Central University.

dswain@nccu.edu

Christopher Cunningham, NC Central University.

chris.cunningham@nccu.edu

Nijee Brown, NC Central University.

nbrow120@eagles.nccu.edu

Building on the vision of MCBK to use shareable and FAIR computable formats to improve human health, we are working on sustainable Open Education Resources (OER). Apply grant funding from the Institute of Museum and Library Sciences (IMLS), we worked with MCBK partners to provide pilot training for 20 students and professionals in health care, library management, and information science (December 15, 2021—January 6, 2022). Using their feedback from pre‐ and post‐assessments, discussions with mentors, and group reports on presentations, we are developing OER modules for the web and a draft for an online, open textbook that will provide sustainable education resources for learners worldwide.

Our “lightning talk” will highlight the presentations that the MCBK pilot training and OER plans require and summarize the leadership recommendations and roles especially relevant to Health Sciences librarians and computer tool developers. The objective is a community of practice (CoP) in design and development CBK collections, repositories, technology, databases, and digital archiving. Librarians, knowledge managers, and information professionals can be strong members of that CoP. Many who participated in the pilot and related meetings are showing interest in learning and demonstrating the skills needed to lead health professionals, researchers, and patients in creating, accessing, and implementing CBK systems.

To enhance academic and professional training, primary learning modules will cover:

1. Learning Health Systems Continuous Cycles (P2D, D2K, and K2P)
2. Open Access publishing instructions and process (LHS journal)
3. Mitigating algorithmic injustice or coded bias, and supporting FAIR and CARE Principles
4. Support for entrepreneurial and open access developers (PATTIE)
5. Effective systematic reviewing (using Academy of Medicine, PICO, and PRISMA)
6. Defining MCBK metadata and how data research networks operate when using CBK technical and infrastructure principles
7. Guiding Trust in behavior and policies for “commons” and knowledge sharing
8. Locating and applying NLM resources and tools (comparing NIH common data elements)

Continuing project contributors to the pilot training and educational resources include: Charles Friedman, Nancy Allee, Kathleen Young, Joshua Richardson, Rachel Richesson, Gerald Perry, Javed Mostafa, Feili Tu‐Keefer, Chris Shaffer, Kate Majewski, Gabe Rios, Will Cross, and Cheryl Casey.

Our research and development team from NC Central University includes: Deborah Swain (project director), Christopher Cunningham (co‐project director), Nijee Brown (MLS graduate assistant), William Tanner (MIS graduate assistant), and Danielle Colbert‐Lewis (librarian and pilot class participant).

In our recorded talk, we will introduce learning materials, recommend reading and videos, and present challenges to librarians, knowledge managers, and information professional to support health professionals, researchers, and patients during the current and future evolution of CBK ecosystems to improve human health worldwide. In our live thematic group discussion, we will invite sharing of health problem research, request suggestions for MCBK training materials, and share our OER updates to support LHS.

## Clinical prediction models for knee pain in patients with knee osteoarthritis: A systematic review and critical appraisal

Beibei Tong, School of Nursing, Peking University, Beijing, China.

2111110230@bjmu.edu.cn

Hongbo Chen, Department of Epidemiology and Biostatistics, School of Public Health, Peking University, Beijing, China.

Cui Wang, School of Nursing, Peking University, Beijing, China.

Wen Zeng, School of Nursing, Peking University, Beijing, China.

Dan Li, School of Nursing, Peking University, Beijing, China.

Peiyuan Liu, School of Nursing, Peking University, Beijing, China.

Ming Liu, Macao Polytechnic University, Macao, China.

Xiaoyan Jin, Macao Polytechnic University, Macao, China.

Shaomei Shang, School of Nursing, Peking University, Beijing, China.

**Study Design:** Systematic review of clinical prediction models.

**Background:** Many prediction models of knee pain have been developed; however, there is considerable heterogeneity among studies. A systematic review summarizing these prediction models for the knee pain may provide an important resource.

**Objectives:** To identify all existing prediction models for the knee pain, and to access model performance, summarize variables in the final models.

**Methods:** A comprehensive literature search was conducted in five databases (PubMed, EMBASE, CINAHL, Web of Science and Cochrane Library) from their inception to March 2022. The key phrases included “knee osteoarthritis” or “KOA” and “predict* tool,” “predict* model,” “algorithm” or “nomogram,” and “knee pain,” “knee complaint*.” The Prediction model Risk of Bias Assessment Tool (PROBAST) was used to assess the quality of the included studies.

**Results:** The final sample contained 16 articles with 26 models for evaluation. The most common model is the onset of knee pain model, followed by knee pain progression model. Only one model was considered strongly predictive (AUC = 0.81), and only three studies externally validated their developed model. In general, models that perform well used larger sample sizes and incorporated comprehensive predictors, such as demographic, clinical, and radiographic variables.

**Conclusion:** Many knee pain prediction models do show promise, although few are externally validated. Cumulative evidence suggests that a combination of demographic, clinical, and radiographic variables may be able to predict knee pain. However, the clinical extensibility, applicability, and interpretability of prediction tools should be considered during model development.

## Operationalizing text‐based Clinical Practice Guidelines as computable decision support on blockchain using code generation

William Van Woensel, University of Ottawa.

wvanwoen@uottawa.ca

Oshani Seneviratne, Rensselaer Polytechnic Institute.

senevo@rpi.edu

Manan Shukla, Rensselaer Polytechnic Institute.

shuklm@rpi.edu

Digital physiological health data, captured by consumer‐grade health sensors with Internet of Medical Things (IoMT) connectivity, allows for timely health alerts in case of adverse events, avoids unnecessary clinic visits for vital measurements, and supports a reliable, longitudinal record of physiological measurements. There is a need for a secure and transparent computational framework to record, integrate, and analyze personal health data to support both patient self‐management (eg, alerts) and assist healthcare providers during patient encounters (eg, summaries, recommendations). Blockchain technology has been studied as a secure and decentralized solution for deploying Electronic Health Records (EHR), as it does not require placing trust in a single provider and avoids fragmentation of longitudinal health profiles across different systems. By further integrating Clinical Decision Support (CDS) features into blockchain technology, which analyze health data to issue health alerts and recommendations, one can pro‐actively support both patients and healthcare providers in their decision making. However, CDS has not received much attention in blockchain.

We aim to mobilize the best available clinical knowledge, found in static and text‐based Clinical Practice Guidelines (CPG), into a form that is computable on blockchain, namely smart contracts. First, the often complex, multi‐step, and declarative decision logic within CPG is captured as logic‐based CDS models (ie, Notation3 ruleset) by knowledge engineers and clinical domain experts. Subsequently, we employ an automated code generation approach to convert the declarative CDS models into directly executable smart contracts. In doing so, we aim to bridge the divide between clinical decision logic and blockchain technology; mobilizing clinical knowledge in a trustworthy and transparent manner to all the stakeholders involved. Our graph‐based code generation approach, implemented as the Smart Contract Generator, includes a Parser that extracts rule graphs from the CDS models; a converter that generates a series of intermediate programming abstractions; and a Code Generator that generates executable blockchain code in an imperative programming language (Solidity). The original CDS model will infer alerts or recommendations as per the encoded clinical decision logic; smart contracts will similarly emit events on blockchain that can be caught by any decentralized web3 application and subsequently acted upon.

To evaluate our approach, we selected several rules from the American Diabetes Standards of Care CPGs, authored the declarative CDS models, and converted them into smart contracts using our Smart Contract Generator. Subsequently, we activated these smart contracts with a relevant subset of the MIMIC‐III dataset. Our evaluation confirms the feasibility of our approach, with insignificant execution times for generated smart contracts, and consistent recommendations between smart contracts and the original CDS models.

This lightning talk will outline the challenges of converting declarative clinical decision logic into a set of smart contracts, shortly describe our Smart Contract Generator, and summarize our evaluation results.

## The right decision service—National decision support for Scotland

Dr. Ann Wales, Digital Health & Care Innovation Centre.

ann.wales@dhi-scotland.com

Implementation and evaluation of the Right Decision Service—the national decision support service for Scotland's health and social care.

Implementation of the Right Decision Service as the national decision support service for Scotland's health and social care involves three interlinked work strands. These will be illustrated through case studies involving three decision support solutions—high risk prescribing decision support embedded in electronic health record systems; a web and mobile decision support toolkit for residential care homes and care at home services; and a suit of web and mobile apps delivering local and national guidelines and pathways.

Work strand one: technology.

Delivery of the Right Decision Service platform as a national set of tools which:

- Convert validated evidence, guidance and best practice into a diverse range of computerized knowledge formats. This includes actionable knowledge summaries, interactive visual pathways, and structured rules (algorithms). Algorithms can be bespoke or written in standards‐based decision support languages—GDL or CQL.
- Store structured and semi‐structured computerized knowledge in a single national repository.
- Deploy, reuse and customize these computerized knowledge entities within web and mobile applications, and integration with electronic health record systems.

The Right Decision platform has delivered over 20 national and local decision support solutions using these tools.

Work strand two: processes.

- A pipeline of clinical safety and quality management processes which provide assurance around safe and effective use of the decision support tools in practice.
- An evaluation framework based on a logic model, setting out short‐, medium‐ and long‐term outcomes and measures.
- An implementation and spread framework being developed to facilitate widespread adoption of the Right Decision Service and the decision support solutions it delivers. This will provide:
- Guidance on roles, process and system change, and implementation methodologies.
- A service model for local and national implementation of decision support, including service goals, components, roles, governance and accountability requirements.

Work strand three: people.

- A workforce development program for knowledge, information and data staff, as key to engineering decision support solutions and facilitating implementation.
- A competency framework and program of learning opportunities to build confidence across the health and social care workforce in designing, interpreting, evaluating and implementing decision support solutions.

## KGRID‐CN, a platform for scaling computable biomedical knowledge across practice in China

Jingyi Wu, Advanced Institute of Information Technology, Peking University, Hangzhou 311 215, China.

joywu@pku.edu.cn

Qing Li, Advanced Institute of Information Technology, Peking University, Hangzhou 311 215, China.

blacknepia@dingtalk.com

Hong'an Pan, Advanced Institute of Information Technology, Peking University, Hangzhou 311 215, China.

panha@aiit.org.cn

Pengfei Li, Advanced Institute of Information Technology, Peking University, Hangzhou 311 215, China.

pfli@aiit.org.cn

Guilan Kong, National Institute of Health Data Science, Peking University, Beijing 100 191, China.

guilan.kong@hsc.pku.edu.cn

Scaling biomedical knowledge across clinical practice is critical to alleviating the contradiction between the growing needs for health services and the limited medical resource. To promote the translation of computable biomedical knowledge (CBK), the Agency for Healthcare Research and Quality in the United States came up with the idea of Learning Health Systems (LHS), the core of which is the virtuous cycle of biomedical data, evidence, and clinical practice. The Knowledge Grid China (KGrid‐CN) platform is a practice of LHS in China, inspired by the Knowledge Grid platform developed by the University of Michigan Medical School. Most CBK in China is dispersed and unexploited in a mass of healthcare systems. In line with China's national conditions of healthcare informatization, KGrid‐CN provides a platform for scaling CBK across practice more quickly, easily, and broadly. Based on the plug‐in architecture, KGrid‐CN regards knowledge objects as the basic unit of data storage and transfer, which could bundle CBK into externalized and callable modules.

The basic framework of KGrid‐CN consists of libraries of knowledge objects, activators of knowledge objects, and the application platform. The workflow of translating specific CBK via KGrid‐CN is as follows. First, the knowledge providers, like healthcare researchers or institutions who can provide the authoritative and latest CBK resources, extract the actionable knowledge and make it machine‐ready. Second, the knowledge translators, those hospitals or healthcare companies devoted to activating CBK as services to generate health advice, transform the CBK into knowledge objects and archive them in the KGrid‐CN libraries. Then, the KGrid‐CN activators are employed to activate and combine the knowledge objects from the libraries and deploy them as an industry solution for a specific practice. These activators could help integrate the fragmented CBK as a comprehensive knowledge system for health services. Based on embedded engines like Nashorn, the KGrid‐CN activators support various execution modes and programming languages. Third, for knowledge users, like physicians or organizations who can apply health advice to real‐world decisions for better outcomes, KGrid‐CN provides them with application programming interface (API) of each knowledge object, as well as a fully visual and interactive operating interface, namely, the application platform, to apply the above industry solutions to different clinical scenarios. Based on the techniques like dynamic configuration loading, KGrid‐CN could automatically generate the visual interface for each application. Finally, with the minimum communication cost, the latest CBK could be translated into clinical practice quickly and easily.

As the first platform mobilizing CBK in China, KGrid‐CN focuses on the field of chronic diseases and has developed a series of implementation standards for scaling CBK across clinical practice. In KGrid‐CN, there have been 29 reusable knowledge objects for various chronic diseases, 14 applications compatible with multiple clinical scenarios, and two special topics for the management of chronic kidney disease. KGrid‐CN has reformed the patterns of CBK management and translation in China and has the potential to decrease the latency between the moment new biomedical knowledge emerges and its widespread uptake and use in practice. This could furtherly help to improve the efficiency and quality of medical service in China.
